# The Impact of Serum Amyloid P-Component on Gene Expression in RAW264.7 Mouse Macrophages

**DOI:** 10.1155/2016/9380290

**Published:** 2016-04-28

**Authors:** Dan Xi, Jinzhen Zhao, Jichen Liu, Haowei Xiong, Wenshuai He, Jing Hu, Wenyan Lai, Zhigang Guo

**Affiliations:** Division of Cardiology, Huiqiao Medical Center, Nanfang Hospital, Southern Medical University, Guangzhou, Guangdong 510515, China

## Abstract

Serum amyloid P-component (SAP) contributes to host defense and prevents fibrosis. Macrophages are the most abundant inflammatory cell type in atherosclerotic plaques. In the present study, using ^3^H-cholesterol-labeled counting radioactivity assay, we demonstrated that the apoAI-mediated cholesterol efflux in RAW264.7 macrophages was increased by SAP treatment in a time- and dose-dependent manner. We analyzed global gene expression changes upon SAP treatment using RNA sequencing. As a result, a total of 175 differentially expressed genes were identified, of which 134 genes were downregulated and 41 genes were upregulated in SAP treated cells compared to control cells. Quantitative RT-PCR analysis confirmed decreased expression of 5 genes and an increase in expression of 1 gene upon SAP treatment. Gene ontology analysis showed that genes involved in response to stimulus were significantly enriched in differentially expressed genes. Beyond protein-coding genes, we also identified 8 differentially expressed long noncoding RNAs. Our study may provide new insights into mechanisms underlying the functional role of SAP in macrophages.

## 1. Introduction

Serum amyloid P-component (SAP) is a member of the pentraxin protein family which was first isolated and identified in “amyloid” pathological deposits. Under normal conditions, SAP is thought to be synthesized and secreted only in hepatocytes. In some diseases, SAP can also be generated by macrophages and smooth muscle cells such as in the atherosclerotic aortic intima [1]. In humans, SAP is constitutively expressed and contributes to host defense through the classical pathway. Studies indicate that SAP does not exist in normal aortic intima but deposits in human atherosclerotic aortic intima and that plasma SAP levels are positively associated with cardiovascular disease [2]. Additionally, SAP binds to amyloid-like structures in oxidized low density lipoprotein (ox-LDL) and prevents lipid uptake by macrophages, suggesting an important role for SAP in atherosclerosis [3]. It will be necessary to further explore the roles of SAP in lipid metabolism and atherosclerosis.

Atherosclerosis has been known as an inflammatory disease for many years. Macrophages are an essential component of the innate immunity and mediate inflammatory responses by recognizing pathogens and producing proinflammatory mediators. Macrophages are the most abundant inflammatory cell type in atherosclerotic plaques. Macrophages are transformed into foam cells upon modified low density lipoprotein uptake and their subsequent death within lesions fuels the formation of the highly proinflammatory and thrombogenic lipid-rich necrotic core [4, 5]. A study revealed that SAP may participate in cholesterol removal from macrophages through its role in promoting cholesterol efflux [6]. The murine macrophage cell line RAW264.7 is easy to propagate and possesses high efficiency for DNA transfection and sensitivity to RNA interference. This cell line is often used in vitro to evaluate the effects of inflammation process [7] in progress of atherosclerosis [8] and especially in cholesterol efflux research [9]. It is a suitable cell line for experiments and our research group has done many experiments using this cell line [10, 11].

By decreasing the numbers of fibrocytes and profibrotic macrophages [12], exogenous administration of SAP has been shown to reduce fibrosis in animal models [13, 14]. Recently, it has also been demonstrated that a type of recombinant human SAP (PRM-151) is able to reduce fibrocytes in pulmonary fibrosis patients [15]. The decreased accumulation of fibrocytes by SAP might be due to reduced leukocyte recruitment via lowering the levels of inflammatory cytokines [16]. Our research group has found that SAP levels significantly increased in acute coronary syndrome (ACS) patients compared with controls [17]. Furthermore, we also revealed that HDL subfractions from ACS patients possess significantly elevated SAP levels, suggesting that SAP may have vital effects on HDL subfraction functions [17]. In the present study, we investigated the effect of SAP on cholesterol efflux in macrophages, and we also attempted to analyze global gene changes associated with RAW264.7 macrophage cells after SAP treatment using RNA sequencing. Our data afforded the opportunity to test the hypothesis that SAP exerts global transcriptional effects on macrophages.

## 2. Materials and Methods

### 2.1. Cell Culture and Treatment

Murine RAW264.7 macrophage cell line was purchased from China Center for Type Culture Collection (CCTCC, Wuhan, China). The RAW264.7 macrophages were seeded in six-well flat bottom culture at 1.0 × 10^6^ cells per well in DMEM (Gibco, Life Technologies, China) containing 10% fetal bovine serum (Gibco, Life Technologies, EU Approved Origin, South America) and maintained at 37°C in a humidified atmosphere of 5% CO_2_. Human serum amyloid P-component (SAP) was purchased from Calbiochem (Calbiochem, EMD Chemicals, MA, USA). SAP was frozen in PBS without the sodium azide preservative. Before experiment, cells were synchronized by changing DMEM supplemented with 2% bovine serum albumin (BSA, Amresco, USA) for 24 h. Then, cells were cultured in primary six-well plates and treated with different concentrations of SAP. BSA served as control.

### 2.2. Assay of apoAI-Mediated Cholesterol Efflux

Murine RAW264.7 macrophage cells were incubated in culture medium containing 30 *μ*g/mL ox-LDL (Yiyuan Biotechnologies, Guangzhou, China) and 1 *μ*Ci/mL [1*α*,2*α*-^3^H]-cholesterol (Amersham Life Science, USA) for 24 h. After being washed with serum-free medium, the cells were incubated in DMEM with 0.2% BSA containing various concentrations of SAP (0~10 *μ*M, 1 *μ*M SAP = 127 mg/mL of SAP pentamers) for another 6 h, 24 h, 48 h, respectively. Cells were subsequently incubated in serum-free medium (without BSA) with or without 10 *μ*g/mL apoAI (Calbiochem, Germany) for 6 h. Then the incubation medium was collected while the cells were washed with PBS and lysed with 0.1 M NaOH. Lastly, the radioactivity of medium and cell lysates was measured by liquid scintillation spectrometry. The cholesterol efflux rate was presented as the ^3^H-cholesterol radioactivity of medium normalized to total ^3^H-cholesterol radioactivity [18].

### 2.3. RNA Sequencing

Before RNA isolation, cells were washed by PBS for three times. Total RNA was isolated using TRIzol reagent (Invitrogen, USA). Samples were sent to Beijing Genomics Institute (BGI) for further bioinformatic analysis. BGI is a genome sequencing center headquartered in Shenzhen, Guangdong Province, China. RNA purity was assessed using the ND-1000 NanoDrop. The* A*260/*A*280 ratio for each RNA sample was greater than 1.8 and the* A*260/*A*230 ratio was greater than 2.0. RNA integrity was evaluated using the Agilent 2100 TapeStation. Only RNA samples with a RINe value above 7.0 were retained. Oligo(dT) magnetic beads were used to isolate poly(A) + mRNAs. The mRNAs were fragmented to approximately 200 bp in fragmentation buffer. Subsequently, these fragments were used as templates for first-strand and second-strand cDNA synthesis. The double-stranded cDNA fragments were purified, end-repaired, and then ligated to sequencing adapters. After purification, suitable fragments were enriched by PCR amplification according to instructions of TruSeq® RNA LT/HT Sample Prep Kit (Illumina). Finally, PCR products were purified and quantified for high-throughput sequencing using the Illumina HiSeq*™* 2500.

### 2.4. Bioinformatic Analysis of RNA Sequencing Data

A computational pipeline was employed to process the raw data from RNA sequencing. Sequence data in fastq format were filtered to remove reads with unknown nucleotides. Clean reads were mapped to mouse reference genome mm9 by using Tophat v1.4.0 [19]. No more than two mismatches were allowed. The mapped reads were assembled into genes and transcripts by Cufflinks v1.3.0 [20]. Gene models were downloaded from the UCSC RefSeq annotation. Gene expression levels were calculated using fragments per kilobase of transcript per million mapped fragments (FPKM) in Cufflinks. Differentially expressed genes were chosen according to the criteria of fold change > 2 and FDR < 0.05. GO enrichment analysis was performed by using BiNGO 2.3 with the GOslim dataset [21]. To test for enrichment, a hypergeometric test was conducted followed by Benjamini and Hochberg multiple test correction. The adjusted *p* value < 0.05 was used as the significance threshold to identify enriched categories. The STRING database v10.0 (http://string-db.org/) was used to create gene network. Connectivity for each gene was analyzed by in-house MATLAB scripts. The connectivity threshold value for hub genes was the mean plus two standard deviations.

### 2.5. Validation by Quantitative RT-PCR

The total RNA was extracted with RNAiso Plus (TaKaRa Biotechnology, Dalian, China). Reverse transcription was performed at 37°C for 15 min followed by 98°C for 5 min using ReverTra Ace qPCR RT Kit (Toyobo, Osaka, Japan). Quantitative PCR was performed using THUNDERBIRD SYBR qPCR Mix (Toyobo, Osaka, Japan) on the Applied Biosystems 7500 (Life Technologies). The program was as follows: 95°C for 60 sec, followed by 40 cycles of 95°C for 15 sec and 60°C for 45 sec. All reactions were run in triplicate. The GAPDH gene was amplified as a reference gene for normalization. Data were analyzed using 2^−ΔΔCt^ method. Primers used in this study were listed in Supplementary Table  1, available online at http://dx.doi.org/10.1155/2016/9380290.

## 3. Results

### 3.1. ApoAI-Mediated Cholesterol Efflux and Global Analysis of Gene Changes

**Figure 1:**
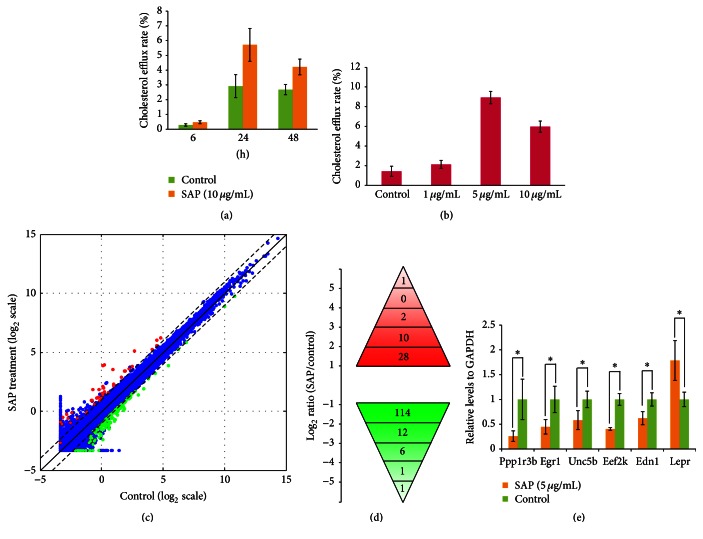
Systematic identification of genes that are altered in SAP treated cells compared to control cells. (a) SAP enhanced apoA1-mediated cholesterol efflux in a time-dependent manner in macrophages. (b) SAP enhanced apoA1-mediated cholesterol efflux in a dose-dependent manner in macrophages for 24 h. (c) Scatter plot depicting the expression profiles of all genes. Log_2_ transformed FPKM values from RNA sequencing were used in the scatter plot. We added 1 to FPKM value before log_2_ transformation to facilitate calculation. Nonchanged genes were shown in blue color while differently expressed genes (fold change > 2 and FDR < 0.05) were denoted in red or green. (d) Distribution of fold change of genes significantly different in SAP treated cells compared to control cells. (e) Validation of RNA sequencing data by quantitative RT-PCR. The GAPDH gene was used as the reference gene for normalization. The statistical significance was tested using unpaired 2-sample* t*-test. Values were plotted as means ± standard error of the mean (SEM) of triplicate measurements. *n* = 3. ^*∗*^ *p* < 0.05.

SAP enhanced apoA1-mediated cholesterol efflux in a time- and dose-dependent manner in macrophages. After 10 *μ*M SAP treatment for 6 h, the cholesterol efflux rate began to rise. The cholesterol efflux rate significantly increased after 24 h compared with 48 h (Figure 1(a)). Compared with control, after SAP treatment for 24 h, 5 and 10 *μ*M SAP significantly increased the cholesterol efflux rate, but there was no significant difference with 1 *μ*M SAP treatment (Figure 1(b)). In order to investigate the molecular mechanism underlying SAP-mediated cholesterol efflux rate increase, transcriptome analysis was performed. For simplicity, cells were treated with 5 *μ*M for 24 h and then subjected to RNA sequencing analysis. We obtained 12,050,636 reads from SAP treated cells and 11,651,186 reads from control cells, respectively. Among all reads obtained in this study, a total of 20,242,256 (85.4%) reads were mappable to the mouse reference genome, of which 79.3% were mapped uniquely to only one location. Absolute gene expression levels were calculated in FPKM (fragments per kilobase of transcript per million mapped fragments) based on RefSeq gene models. We then compared the relative gene abundance in SAP treated cells and control cells. Differentially expressed genes were identified according to their fold changes (>2) and false discovery rate (FDR) adjusted* p* values (<0.05) (Figure 1(c)). Compared to control, 134 genes were downregulated and 41 genes were upregulated after SAP treatment (Supplementary Table 2). The range of fold changes was 41.01~2.00 and 54.14~2.01 for downregulated and upregulated genes, respectively (Figure 1(d)). In order to validate RNA-seq data, we randomly picked up 6 genes for qRT-PCR analysis. The expression trend of these genes measured by qRT-PCR was consistent with RNA sequencing data (Figure 1(e)), suggesting that our RNA-seq data are of high quality.

### 3.2. Gene Ontology (GO) Analysis

**Figure 2:**
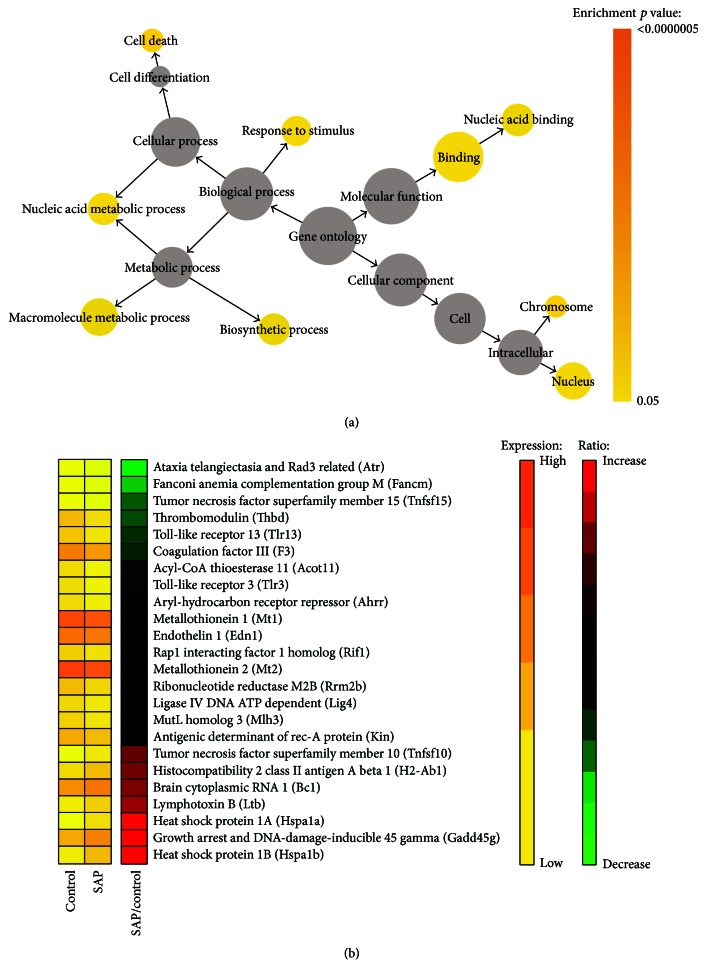
Functional clustering analysis of differentially expressed genes. (a) Differentially expressed genes were analyzed using BiNGO software. Significantly enriched GOslim categories were highlighted with different colors representing different levels of significance. The size of each circle is correlated to the number of genes. (b) Heat map of the 24 differentially expressed genes that fall into the response to stimulus category. In the heat map, the first and second columns correspond to the absolute gene expression levels (FPKM values) of SAP and control group, respectively. Values are color-coded, with yellow representing low levels and orange representing high levels. The third column of the heat map reports the relative expression levels. The values are the log_2_ ratio of SAP versus control. Red indicates increase and green indicates decrease.

All differentially expressed genes (134 downregulated and 41 upregulated) were functionally categorized based on gene ontology (GO) annotation terms using BiNGO software. Enrichment analysis revealed that a total of 9 GO terms exhibited significance as overrepresented terms (*p* < 0.05). In the biological process category, 5 GO terms, namely, biosynthetic process, macromolecule metabolic process, nucleic acid metabolic process, response to stimulus, and cell death, were found to be significantly enriched. GO terms related to cell chromosome and nucleus were significantly enriched under the cellular component category. Enriched GO terms in the molecular function category were binding and nucleic acid binding. The hierarchical organization of these GO terms is shown in Figure 2(a), together with the significance of enrichment indicated by different colors. Strikingly, a total of 24 differentially expressed genes fell into the response to stimulus category. Included were 17 downregulated genes (Atr, Fancm, Tnfsf15, Thbd, Tlr13, F3, Acot11, Tlr3, Ahrr, Mt1, Edn1, Rif1, Mt2, Rrm2b, Lig4, Mlh3, and Kin) and 7 upregulated genes (Tnfsf10, H2-Ab1, Bc1, Ltb, Hspa1a, Gadd45g, and Hspa1b) (Figure 2(b)).

### 3.3. Network Analysis of SAP-Regulated Genes

**Figure 3:**
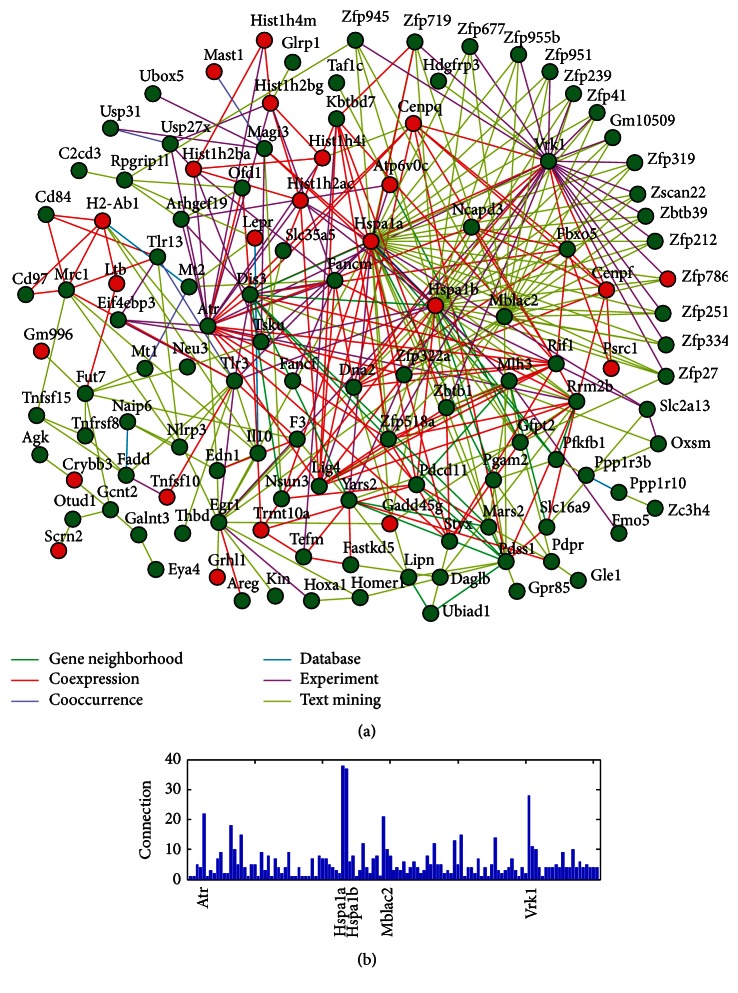
Network analysis of SAP-regulated genes. (a) The gene network generated by STRING. (b) Bar plot showing connection degrees for all genes.

Network analysis can help understand the molecular and cellular interactions. It can be visualized to represent genes (nodes) and their relationships (edges). In the present study, we investigated functional interaction among SAP-regulated genes using the web-based network tool STRING (http://string-db.org/). The results from STRING were shown as Figure 3(a). The highly connected nodes, also known as hub genes, represent functionally important genes in the network. Connectivity analysis showed that Atr, Hspa1a, Hspa1b, Mblac2, and Vrk1 were hubs of the network (Figure 3(b)). Additionally, 4 genes (Tnfsf15, Tlr3, Nlrp3, and Lepr), which have known roles in cholesterol efflux and atherosclerosis, were present in the network.

### 3.4. Altered Expression of Noncoding RNA Genes

**Figure 4:**
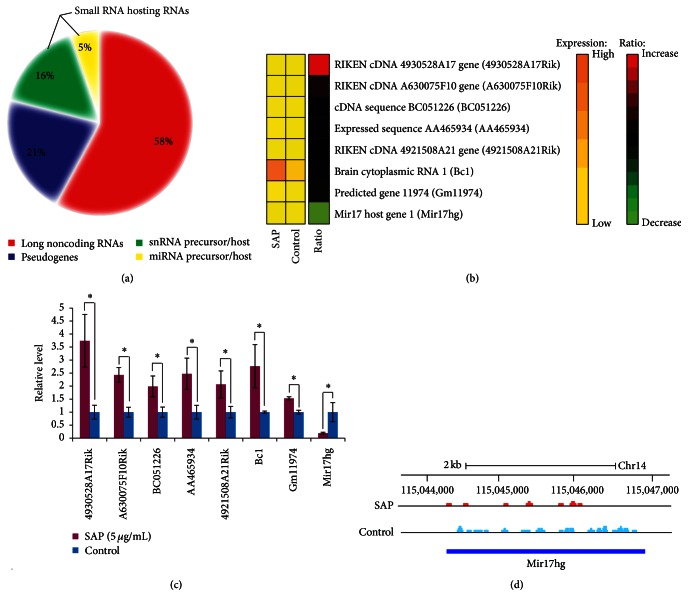
Changed expression of noncoding RNA genes. (a) Classification of noncoding RNA genes. Pie chart is displayed on the differently expressed 19 noncoding RNA genes. (b) Heat map of the 8 selected long noncoding RNA genes. The first and second columns correspond to the absolute gene expression levels (FPKM values) in SAP group and control group, respectively. The third column of the heat map reports the relative expression levels. Values are color-coded as indicated by the color bars. (c) Quantitative RT-PCR analysis of 8 selected lncRNAs. The GAPDH gene was used as the reference gene for normalization. Statistical significance was tested using unpaired 2-sample* t*-test. Values were plotted as means ± standard error of the mean (SEM) of triplicate measurements. *n* = 3. ^*∗*^ *p* < 0.05. (d) RNA sequencing coverage plot of Mir17hg lncRNA. Coverage plot is displayed on the reference genome (UCSC mm9). The upper panel represents expression in SAP group and the lower panel represents expression in control group. For both panels, numbers on *y*-axis refer to RNA sequencing read-depth at a given nucleotide position.

Although the purpose of the present study was to measure polyadenylated mRNAs, it was possible that additional polyadenylated noncoding RNA genes might be present in our dataset. We examined all the 175 differentially expressed genes and discovered 19 noncoding RNA genes, including 11 long noncoding RNAs, 4 pseudogenes, 3 snRNA precursor/hosting RNAs, and 1 miRNA precursor/hosting RNA (Figure 4(a)). Among them, we validated the expression levels of 8 noncoding genes (Figure 4(b)). The expression trend of these genes measured by qRT-PCR was consistent with RNA sequencing data (Figure 4(c)). Strikingly, the miRNA hosting gene Mir17hg is significantly downregulated which is resided on the plus strand of chromosome 14 (Figure 4(d)).

## 4. Discussion

Serum concentration of SAP in normal humans is about 30~40 *μ*g/mL. In healthy mice, normal SAP levels may be as low as 10 *μ*g/mL in the C57BL strain and as high as 100 *μ*g/mL (approximately 1 *μ*M) in the DBA/2 strain [22]. In the present study, RAW264.7 mouse macrophages were used. Therefore, we chose 1 *μ*M to represent a physiological situation. Higher concentrations up to 10 *μ*M were used to mimic the acute phase reaction. We showed that SAP treatment induced macrophage cholesterol efflux with a time- and dose-dependent manner. The transcriptomic differences upon SAP treatment were determined by using RNA sequencing (RNA-seq). RNA-seq is an unbiased method which is not limited to detecting predesigned sequences [23]. In contrast to microarray, RNA-seq does not suffer from cross-hybridization [24]. Additionally, RNA-seq does not have any upper limit for quantification, making it a highly accurate tool for quantifying gene expression levels.

Through RNA sequencing, we identified a total of 175 differentially expressed genes, of which 134 genes were downregulated and 41 genes were upregulated after SAP treatment. Among these genes, 14 genes (6 protein-coding genes and 8 noncoding genes) were selected and validated by using qRT-PCR. In general, the expression trend of these genes measured by qRT-PCR was consistent with RNA sequencing, suggesting that our data were of high quality. Based on gene ontology analysis, a total of 9 GO terms exhibited significance as overrepresented terms: biosynthetic process, macromolecule metabolic process, nucleic acid metabolic process, response to stimulus, and cell death in the biological process category; cell chromosome and nucleus under the cellular component category; and binding and nucleic acid binding in the molecular function category. These results suggest that SAP treatment may have a wide effect on macrophages.

In our present study, we found that TNFSF15, TLR3, and NLRP3 were downregulated upon SAP treatment. Tumor necrosis factor superfamily member 15 (TNFSF15), also known as vascular endothelial growth inhibitor (VEGI) or TNF ligand related molecule 1A (TL1A), is a unique cytokine that functions as a modulator of vascular homeostasis and inflammation [25, 26]. TNFSF15 is involved in numerous cellular processes including the suppression of neovascularization which is essential for tumor progression and spread [26, 27]. TNFSF15 inhibits cholesterol efflux and suppresses the expression of three proteins, apoE, ABCA1,  and ABCG1, both in vitro and in vivo [28, 29]. Toll-like receptors (TLRs) are the most characterized innate immune receptors as well as pattern-recognition receptors (PRRs) [30, 31]. Scavenger receptors induced by TLR3 could regulate increased lipid uptake and activated TLR3 also increases TG accumulation in RAW cells [32, 33]. Indeed, stimulation of TLR3 or TLR4 by pathogen-derived ligands inhibits expression of LXR-dependent gene targets and macrophage cholesterol efflux via a MyD88-independent mechanism and involves IRF3 [34, 35]. The nucleotide-binding domain, leucine-rich-containing family, pyrin domain-containing-3 (NLRP3) inflammasome has emerged as an important regulator of inflammation in metabolic disorders and atherosclerosis [36, 37]. Recent study also showed abnormal lipid deposition and lysosomal cholesterol accumulation are due to impaired intracellular lipid trafficking in macrophages upon Nlrp3 inflammasome activation by nonatherogenic stimulus ATP [38]. Moreover, mRNA levels of ABCA1 and ABCG1 were increased after the treatment of NLRP3i, suggesting that NLRP3 gene silencing could be a potentially therapeutic mean to increase macrophage cholesterol efflux for the prevention of atherosclerosis [39]. Therefore, elevated cholesterol efflux in macrophages by SAP may be mediated by the repression of TNFSF15, TLR3, and NLRP3.

Lepr, the leptin receptor, is expressed in many tissues including the cardiovascular system [40]. HDL-mediated cholesterol efflux was suppressed by leptin and expression of long form of Lepr was upregulated during monocytic differentiation into macrophages and sustained after differentiation [41]. In this study, we found that Lepr was upregulated upon SAP treatment, suggesting that SAP may also elevate cholesterol efflux via the upregulation of Lepr.

Although emerging evidence indicates long noncoding RNAs (lncRNAs) may have functional significance in development, physiology, and diseases [42], they remain unexplored on macrophage research. In this study, we identified 19 differentially expressed lncRNAs. Previously, the microRNA 17-92 cluster host gene (MIR17HG) has been shown to regulate expression of genes involved in breast cancer development and progression [43]. The functions of the other lncRNAs, including 6330549D23Rik, 4930528A17Rik, BC051226, AA465934, 4921508A21Rik, and Gm11974, have not been determined yet. Notably, SAP-induced genes such as Gadd45g are associated with cancer [44]. The idea that long-term use of SAP in patients might promote tumorigenesis deserves further investigation.

In conclusion, in the present study we found that SAP treatment increased cholesterol efflux rate. We analyzed global gene changes upon SAP treatment in RAW264.7 macrophages using RNA-seq. Our study may provide new insights into the molecular mechanisms underlying the functional role of SAP in macrophages.

## Supplementary Material

Supplementary Table 1. Primers used in this study for quantitative RT-PCR analysis.
Supplementary Table 2. The complete gene list of differentially expressed genes (fold change > 2 and FDR < 0.05).
