# Supplementary material for: The Impact of Serum Amyloid P-Component on Gene Expression in RAW264.7 Mouse Macrophages
Source: Biomed Res Int. 2016 Apr 28;2016:9380290. doi: 10.1155/2016/9380290 (PMC4864538; doi:10.1155/2016/9380290)
Supplement: Supplementary file 1 — Supplementary Table 1. Primers used in this study for quantitative RT-PCR analysis. Supplementary Table 2. The complete gene list of differentially expressed genes (fold change > 2 and FDR < 0.05). [file 9380290.f1.pdf]

Supplementary Table 1. Primers used in this study for quantitative RT-PCR analysis.

Symbol	Gene ID	Primers	Product
Edn1	NM_010104.3	gctggtggaaggaaggaaac;agtcaatgtgctcggttgtg	132
Egr1	NM_007913.5	ccttcagcacctcaactggt;tctttcctgcctctcccttt	108
Nlrp3	NM_145827.3	cctttgacgagcacattgg;cttgggcagcagtttctttc	101
Il10	NM_010548.2	aaataagagcaaggcagtgg;gtccagcagactcaatacacac	171
Ppp1r3b	NM_177741.3	tacccagcagcctcagaaga;atgcctatcccagacacgag	141
Unc5b	NM_029770.2	ccagactcctacccatcagc;gacccactcgccattacact	153
Pgam2	NM_018870.3	gctgagtgagaagggagcag;gtccaaagggtgcggatt	120
Eef2k	NM_001267710.1	aatggctgtttctccttcca;atcctgtcggcttgttcttg	105
Lepr	NM_001122899.1	agccacgcaacttctccag;aaaccaacccaccctctttc	101
4930528A17Rik	NR_028384.1	tgtctgtgtgagggtgtcaaat;tggtggaaactgggagtca	154
A630075F10Rik	NR_033632.1	caagagaggcaggcaagaag;atggagagtgtgagccaagg	137
BC051226	NR_045146.1	gtgttgatgaggaagcctgaa;acaatgagaggaggtgggttta	165
AA465934	NR_028363.1	attgtccctgtccaagactgc;tttcaaccctgatttctcacct	187
4921508A21Rik	NR_046034.1	ctggaaccctgaactgacaac;tatgtggagagattgctggaga	139
Gm11974	NR_045893.1	ggctattccgagtccaggta;gtcatcccttccacgctgta	147
6330549D23Rik	NR_003619.2	tggaaggttctccaagatcagt;gtcttcgtgctccctaagtctg	102
Mir17hg	NR_029382.1	tgaagatgccgatttccact;atccagcgagcaaacacg	186
Bc1	NR_038088.1	gggatttagctcagtggtagag;ggttgtgtgtgccagttacc	144

Supplementary Table 2. The complete gene list of differentially expressed genes (fold change > 2 and FDR < 0.05).

Symbol	SAP	Control	log2 ratio	pvalue	FDR	Title
Fbxo5	0.01	0.41	-5.36	0.0071884	0.03263802	F-box protein 5
Zfp951	0.01	0.26	-4.68	0.0071884	0.0326487	zinc finger protein 951
Ccdc96	0.03	0.42	-3.84	0.0004479	0.00305774	coiled-coil domain containing 96
Pfkfb1	0.06	0.87	-3.84	0.0004479	0.00305924	6-phosphofructo-2-kinase /fructose-2,6-biphosphatase 1
Armcx2	0.03	0.37	-3.73	0.0008518	0.00533769	armadillo repeat containing, X-linked 2
Mab21l3	0.03	0.33	-3.49	0.0030434	0.01594675	mab-21-like 3 (C. elegans)
Fut7	0.05	0.54	-3.35	0.0057106	0.02691448	fucosyltransferase 7
Arhgef19	0.03	0.28	-3.20	0.0106515	0.04584291	Rho guanine nucleotide exchange factor (GEF) 19
Gpr19	0.10	0.62	-2.61	0.006566	0.03027786	G protein-coupled receptor 19
Atl1	0.19	1.06	-2.52	2.99E-05	0.00027365	atlastin GTPase 1
6330549D23Rik	0.07	0.41	-2.49	0.011622	0.04931605	RIKEN cDNA 6330549D23 gene
Usp27x	0.10	0.53	-2.44	0.0022189	0.01214902	ubiquitin specific peptidase 27, X chromosome
Apba1	0.06	0.30	-2.28	0.001301	0.0076909	amyloid beta (A4) precursor protein binding, family A, member 1
Pgam2	0.35	1.53	-2.15	0.0112975	0.04821917	phosphoglycerate mutase 2
Fmo5	0.06	0.26	-2.15	0.0112975	0.048234	flavin containing monooxygenase 5
Zfp677	0.14	0.62	-2.12	0.0037355	0.01884049	zinc finger protein 677
Atr	0.05	0.22	-2.12	0.0037355	0.01883364	ataxia telangiectasia and Rad3 related
Slc2a13	0.24	0.99	-2.05	3.70E-08	5.79E-07	solute carrier family 2 (facilitated glucose transporter), member 13
Fancm	0.07	0.27	-2.03	0.0020993	0.01159502	Fanconi anemia, complementation group M
Hoxa1	0.21	0.86	-2.03	0.0020993	0.0115858	homeobox A1
Eif3j2	0.17	0.66	-1.94	0.0103715	0.04480436	eukaryotic translation initiation factor 3, subunit J2
Zfp239	0.26	0.98	-1.92	0.0006626	0.00428332	zinc finger protein 239
Mir17hg	0.40	1.50	-1.90	0.0001302	0.00101941	Mir17 host gene 1 (non-protein coding)
Zscan22	0.56	2.08	-1.89	1.88E-06	2.21E-05	zinc finger and SCAN domain containing 22
Tsku	0.19	0.70	-1.88	0.0056785	0.02678156	tsukushi
Glrp1	0.27	0.93	-1.80	0.0092166	0.04052142	glutamine repeat protein 1

Fancf	0.62	2.14	-1.80	0.0001889	0.00142144	Fanconi anemia, complementation group F
Tnfsf15	0.09	0.31	-1.80	0.0092166	0.04050859	tumor necrosis factor (ligand) superfamily, member 15
Zfp719	0.33	1.13	-1.76	3.90E-06	4.35E-05	zinc finger protein 719
Thbd	1.04	3.50	-1.75	2.21E-12	5.65E-11	thrombomodulin
E430018J23Rik	0.39	1.28	-1.73	0.001529	0.00884655	RIKEN cDNA E430018J23 gene
Pdss1	0.51	1.69	-1.73	0.001529	0.00885024	prenyl (solanesyl) diphosphate synthase, subunit 1
Eya4	0.15	0.49	-1.69	0.0080265	0.03590344	eyes absent 4 homolog (Drosophila)
Ppp1r3b	0.44	1.44	-1.69	3.08E-06	3.50E-05	protein phosphatase 1, regulatory (inhibitor) subunit 3B
Lrrc14	1.15	3.61	-1.65	9.17E-08	1.34E-06	leucine rich repeat containing 14
Tlr13	0.81	2.50	-1.63	4.88E-09	8.49E-08	toll-like receptor 13
G630090E17Rik	0.47	1.44	-1.61	0.0037807	0.0190062	RIKEN cDNA G630090E17 gene
Magi3	0.17	0.53	-1.61	0.0006426	0.00417748	membrane associated guanylate kinase, WW and PDZ domain containing 3
Gpr85	0.49	1.51	-1.61	0.0001127	0.00089251	G protein-coupled receptor 85
Egr1	2.62	7.98	-1.61	9.82E-20	4.03E-18	early growth response 1
Zfp41	0.33	1.00	-1.57	0.0005532	0.00368102	zinc finger protein 41
F3	14.47	42.85	-1.57	9.15E-59	1.01E-56	coagulation factor III
Gdpgp1	0.53	1.58	-1.56	2.67E-05	0.00024579	GDP-D-glucose phosphorylase 1
Flrt3	3.66	10.79	-1.56	3.19E-36	2.13E-34	fibronectin leucine rich transmembrane protein 3
Otud1	0.66	1.94	-1.55	8.39E-05	0.0006879	OTU domain containing 1
2610015P09Rik	0.20	0.59	-1.54	0.0107472	0.04621154	RIKEN cDNA 2610015P09 gene
Gfpt2	0.42	1.18	-1.49	0.0012919	0.00764328	glutamine fructose-6-phosphate transaminase 2
Naif1	0.46	1.28	-1.47	0.0006117	0.0040008	nuclear apoptosis inducing factor 1
Lin52	1.27	3.48	-1.46	3.37E-06	3.81E-05	lin-52 homolog (C. elegans)
Unc5b	0.46	1.24	-1.42	7.46E-06	7.71E-05	unc-5 homolog B (C. elegans)
Mblac2	0.27	0.71	-1.41	0.006327	0.02933177	metallo-beta-lactamase domain containing 2
Fastkd5	0.50	1.28	-1.37	0.0011513	0.00690301	FAST kinase domains 5
Zfp27	0.41	1.06	-1.35	0.0020446	0.01133326	zinc finger protein 27
Zbtb1	0.68	1.73	-1.35	2.39E-05	0.00022221	zinc finger and BTB domain containing 1
Neu3	0.69	1.76	-1.35	0.0001035	0.00082741	neuraminidase 3
Zfp334	0.21	0.53	-1.35	0.0094876	0.04150256	zinc finger protein 334
Gm10509	0.68	1.70	-1.33	0.0036374	0.01842616	zinc finger protein 51 pseudogene

Slc35a5	0.97	2.44	-1.33	3.77E-07	4.97E-06	solute carrier family 35, member A5
Tefm	0.88	2.21	-1.33	0.0078474	0.03523823	transcription elongation factor, mitochondrial
Mars2	0.40	1.00	-1.33	0.0078474	0.03524963	methionine-tRNA synthetase 2 (mitochondrial)
Acot11	0.46	1.15	-1.32	6.07E-05	0.00051694	acyl-CoA thioesterase 11
Yars2	1.05	2.62	-1.32	0.0014159	0.00829249	tyrosyl-tRNA synthetase 2 (mitochondrial)
Cd97	0.39	0.95	-1.30	0.0064847	0.02997257	CD97 antigen
Hdgfrp3	0.57	1.40	-1.30	8.26E-06	8.44E-05	hepatoma-derived growth factor, related protein 3
Zfp945	0.28	0.69	-1.29	0.0009775	0.00601383	zinc finger protein 945
Nsun3	1.40	3.41	-1.29	0.0009775	0.0060165	NOL1/NOP2/Sun domain family member 3
Eef2k	0.34	0.84	-1.28	0.0003859	0.0026833	eukaryotic elongation factor-2 kinase
Ncapd3	0.40	0.97	-1.28	0.0003859	0.00268196	non-SMC condensin II complex, subunit D3
Tnfrsf8	1.06	2.57	-1.28	0.0020715	0.01147332	tumor necrosis factor receptor superfamily, member 8
Pdpr	1.22	2.94	-1.26	8.32E-06	8.48E-05	pyruvate dehydrogenase phosphatase regulatory subunit
2310015A10Rik	0.32	0.77	-1.25	0.0036486	0.01847605	RIKEN cDNA 2310015A10 gene
Tlr3	0.43	1.03	-1.25	0.0014199	0.00830853	toll-like receptor 3
Usp31	0.51	1.21	-1.24	1.11E-07	1.60E-06	ubiquitin specific peptidase 31
Exoc8	0.41	0.95	-1.22	8.76E-05	0.00071612	exocyst complex component 8
Galnt3	1.70	3.88	-1.19	3.65E-08	5.71E-07	UDP-N-acetyl-alpha-D -galactosamine:polypeptide N-acetylgalactosaminyltransferase 3
Pdcd11	1.19	2.71	-1.19	3.58E-09	6.31E-08	programmed cell death 11
Ercc6l2	0.49	1.11	-1.19	0.000312	0.00221274	excision repair cross-complementing rodent repair deficiency, complementation group 6 like 2
Lipn	0.90	2.06	-1.19	0.0113393	0.04833838	lipase, family member N
Areg	2.26	5.16	-1.19	0.000312	0.00221161	amphiregulin
Mir22hg	9.05	20.53	-1.18	2.22E-18	8.37E-17	Mir22 host gene (non-protein coding)
Kbtbd7	0.76	1.72	-1.18	5.89E-05	0.00050212	kelch repeat and BTB (POZ) domain containing 7
Fadd	1.24	2.79	-1.17	4.88E-05	0.00042247	Fas (TNFRSF6)-associated via

						death domain
Ahrr	0.55	1.24	-1.17	0.0005357	0.00357479	aryl-hydrocarbon receptor repressor
Ppfibp2	0.54	1.22	-1.17	0.0019933	0.01111112	PTPRF interacting protein, binding protein 2 (liprin beta 2)
Agk	1.22	2.73	-1.16	0.0004425	0.00303011	acylglycerol kinase
Rpgrip1l	0.24	0.54	-1.16	0.0062316	0.02895702	Rpgrip1-like
Cd84	0.47	1.06	-1.16	0.0062316	0.02894733	CD84 antigen
Hdhd3	2.09	4.66	-1.16	0.0013523	0.00795682	haloacid dehalogenase-like hydrolase domain containing 3
Eid2	1.53	3.37	-1.14	0.0034433	0.01762279	EP300 interacting inhibitor of differentiation 2
Zfp322a	0.43	0.93	-1.13	0.0028286	0.01494496	zinc finger protein 322A
Gcnt2	1.43	3.14	-1.13	1.96E-07	2.72E-06	glucosaminyl (N-acetyl) transferase 2, I-branching enzyme
Slc16a9	1.41	3.08	-1.13	3.55E-06	3.99E-05	solute carrier family 16 (monocarboxylic acid transporters), member 9
Ubox5	0.56	1.23	-1.13	0.0023247	0.01265873	U box domain containing 5
Mt1	475.98	1036.26	-1.12	1.05E-257	9.70E-255	metallothionein 1
Zfp518a	0.35	0.76	-1.12	0.0015724	0.00906369	zinc finger protein 518A
Dna2	1.19	2.59	-1.12	5.96E-06	6.34E-05	DNA replication helicase 2 homolog (yeast)
Sacs	0.50	1.08	-1.12	0.0088686	0.03921509	sacsin
Krt23	1.67	3.62	-1.11	0.0010654	0.00647713	keratin 23
Ofd1	0.45	0.96	-1.10	0.0048656	0.02361367	oral-facial-digital syndrome 1 gene homolog (human)
Ppp1r10	1.21	2.59	-1.10	5.69E-06	6.09E-05	protein phosphatase 1, regulatory subunit 10
Mrc1	0.78	1.68	-1.10	4.31E-05	0.00037855	mannose receptor, C type 1
Zfp212	1.50	3.17	-1.08	7.23E-05	0.00060252	Zinc finger protein 212
Edn1	55.87	118.45	-1.08	4.33E-113	1.13E-110	endothelin 1
Vrk1	0.77	1.63	-1.08	0.0008308	0.00522264	vaccinia related kinase 1
Rif1	0.87	1.84	-1.08	9.33E-08	1.36E-06	Rap1 interacting factor 1 homolog (yeast)
Mt2	861.44	1811.87	-1.07	0	0	metallothionein 2
Nlrp3	6.57	13.82	-1.07	1.36E-23	6.54E-22	NLR family, pyrin domain containing 3
Zfp955b	0.55	1.15	-1.07	0.0102229	0.04432806	zinc finger protein 955B
C2cd3	0.92	1.94	-1.07	1.86E-07	2.59E-06	C2 calcium-dependent domain containing 3
Bcl2l12	1.92	4.01	-1.07	0.0055899	0.02643537	BCL2-like 12 (proline rich)
Tbcc	2.32	4.86	-1.06	6.83E-05	0.00057285	tubulin-specific chaperone C

Rrm2b	1.50	3.13	-1.06	5.38E-07	6.89E-06	ribonucleotide reductase M2 B (TP53 inducible)
Il10	37.43	77.64	-1.05	1.50E-40	1.19E-38	interleukin 10
Zc3h4	1.16	2.41	-1.05	4.21E-07	5.47E-06	zinc finger CCCH-type containing 4
Zbtb39	0.82	1.70	-1.05	3.00E-05	0.00027402	zinc finger and BTB domain containing 39
Homer1	1.60	3.29	-1.04	3.28E-07	4.36E-06	homer homolog 1 (Drosophila)
Zfp251	1.01	2.06	-1.03	0.0004952	0.00333473	zinc finger protein 251
Lig4	0.64	1.31	-1.03	0.0028724	0.01514776	ligase IV, DNA, ATP-dependent
Calhm2	1.07	2.19	-1.03	0.0063654	0.02949009	calcium homeostasis modulator 2
Daglb	0.61	1.24	-1.03	0.009521	0.04163563	diacylglycerol lipase, beta
Naip6	0.42	0.86	-1.03	0.001937	0.01083639	NLR family, apoptosis inhibitory protein 6
Kctd21	1.44	2.94	-1.03	0.0001288	0.0010094	potassium channel tetramerisation domain containing 21
Oxsm	0.67	1.36	-1.03	0.003501	0.01784561	3-oxoacyl-ACP synthase, mitochondrial
Mlh3	0.77	1.57	-1.03	0.000156	0.00119749	mutL homolog 3 (E coli)
Taf1c	0.71	1.44	-1.03	0.0052115	0.02496896	TATA box binding protein (Tbp)-associated factor, RNA polymerase I, C
Klhl42	0.30	0.62	-1.03	0.009521	0.04162251	kelch-like 42
Styx	1.23	2.50	-1.03	2.32E-05	0.00021686	serine/threonine/tyrosine interaction protein
Zfp319	2.82	5.74	-1.02	2.98E-10	6.00E-09	zinc finger protein 319
Ubiad1	2.15	4.34	-1.02	3.96E-06	4.39E-05	UbiA prenyltransferase domain containing 1
Kin	3.26	6.57	-1.01	0.00012	0.000944	antigenic determinant of rec-A protein
Gle1	1.07	2.14	-1.01	0.0010002	0.00612102	GLE1 RNA export mediator (yeast)
Eif4ebp3	2.72	5.47	-1.01	0.0014775	0.00859884	eukaryotic translation initiation factor 4E binding protein 3
Dis3	0.76	1.52	-1.00	0.0026625	0.01423007	DIS3 mitotic control homolog (S. cerevisiae)
Tmem177	0.79	1.58	-1.00	0.0039537	0.01975427	transmembrane protein 177
4930528A17Rik	0.54	0.01	5.76	0.0005525	0.00367819	RIKEN cDNA 4930528A17 gene
Mir1938	14.62	1.07	3.78	0.0005999	0.00394471	microRNA 1938
Snord14e	14.92	1.17	3.67	0.0011178	0.00673414	small nucleolar RNA, C/D box 14E
A630075F10Rik	0.71	0.11	2.67	0.004743	0.02308295	RIKEN cDNA A630075F10 gene
Rn7sk	11.64	1.93	2.59	1.32E-06	1.58E-05	RNA, 7SK, nuclear

Scrn2	0.82	0.14	2.56	0.0082938	0.03696805	secernin 2
Hspa1b	3.49	0.61	2.52	1.70E-14	5.18E-13	heat shock protein 1B
Cenpf	0.16	0.03	2.47	0.0017439	0.00988388	centromere protein F
Gadd45g	34.30	6.51	2.40	7.15E-48	6.66E-46	growth arrest and DNA-damage -inducible 45 gamma
Hspa1a	0.93	0.19	2.29	0.0002395	0.00175885	heat shock protein 1A
Zfp786	0.66	0.15	2.14	0.0030462	0.01594928	zinc finger protein 786
Mast1	0.47	0.11	2.11	0.0011028	0.00666674	microtubule associated serine /threonine kinase 1
BC051226	3.59	0.89	2.02	0.0002433	0.00177926	cDNA sequence BC051226
AA465934	4.30	1.10	1.97	0.0082667	0.03685891	expressed sequence AA465934
Hist1h2ac	3.98	1.11	1.84	2.61E-10	5.27E-09	histone cluster 1, H2ac
Ltb	1.86	0.57	1.71	0.0069562	0.03179174	lymphotoxin B
4921508A21Rik	1.93	0.60	1.69	0.0005002	0.00336512	RIKEN cDNA 4921508A21 gene
Lepr	0.51	0.17	1.56	0.0031397	0.01634633	leptin receptor
Bc1	64.43	22.16	1.54	2.50E-08	3.99E-07	brain cytoplasmic RNA 1
Rn45s	10.60	3.69	1.52	4.16E-98	9.02E-96	45S pre-ribosomal RNA
Rogdi	2.18	0.77	1.51	0.0027503	0.01459826	rogdi homolog (Drosophila)
Cx3cr1	2.28	0.82	1.47	5.78E-07	7.36E-06	chemokine (C-X3-C) receptor 1
Psrc1	2.88	1.04	1.47	0.0001422	0.0011028	proline/serine-rich coiled-coil 1
Hist1h2ba	10.61	3.89	1.45	0.0007056	0.00451933	histone cluster 1, H2ba
H2-Ab1	3.15	1.16	1.44	0.0012086	0.00720299	histocompatibility 2, class II antigen A, beta 1
Mir703	74.74	28.37	1.40	2.69E-06	3.09E-05	microRNA 703
Hist1h4m	12.12	4.74	1.35	0.0003946	0.00273435	histone cluster 1, H4m
Tnfsf10	0.65	0.26	1.34	0.004535	0.02223448	tumor necrosis factor (ligand) superfamily, member 10
Atp6v0c	4.90	1.95	1.33	0.0001728	0.00131337	ATPase, H+ transporting, lysosomal V0 subunit C
Hist1h2bg	13.93	5.55	1.33	3.68E-06	4.13E-05	histone cluster 1, H2bg
3000002C10Rik	2.61	1.08	1.27	0.0056251	0.02658363	glyceraldehyde-3-phosphate dehydrogenase pseudogene
Gm11974	7.23	3.06	1.24	0.0020219	0.01124831	predicted gene 11974
Crybb3	4.52	2.00	1.18	0.0048665	0.02360968	crystallin, beta B3
Fam72a	1.49	0.66	1.18	0.0116295	0.04933305	family with sequence similarity 72, member A
Grhl1	0.96	0.43	1.16	0.0097646	0.04254015	grainyhead-like 1 (Drosophila)
Hist1h4i	37.54	17.08	1.14	1.12E-10	2.40E-09	histone cluster 1, H4i
Gm6756	2.20	1.01	1.12	0.0048635	0.02361173	3-phosphoglycerate dehydrogenase pseudogene
Trmt10a	1.86	0.89	1.06	0.0003701	0.00258755	tRNA methyltransferase 10A
Gm996	0.89	0.43	1.04	0.0078977	0.03543007	predicted gene 996
Rnu6	46.85	22.92	1.03	0.0038964	0.01948902	U6 small nuclear RNA

Cenpq	2.97	1.48	1.01	0.0090859	0.04001042	centromere protein Q
